# Hard-Carbon Negative Electrodes from Biomasses for Sodium-Ion Batteries

**DOI:** 10.3390/molecules28104027

**Published:** 2023-05-11

**Authors:** Bin Lu, Chengjun Lin, Haiji Xiong, Chi Zhang, Lin Fang, Jiazhou Sun, Ziheng Hu, Yalong Wu, Xiaohong Fan, Guifang Li, Jile Fu, Dingrong Deng, Qihui Wu

**Affiliations:** 1 College of Marine Equipment and Mechanical Engineering, Xiamen Key Lab of Marine Corrosion and Smart Protective Materials, Jimei University, Xiamen 361021, China; 2 School of Energy and Chemical Engineering, Xiamen University Malaysia, Sepang 43900, Malaysia

**Keywords:** hard carbon, sodium-ion battery, biomass, atom doped

## Abstract

With the development of high-performance electrode materials, sodium-ion batteries have been extensively studied and could potentially be applied in various fields to replace the lithium-ion cells, owing to the low cost and natural abundance. As the key anode materials of sodium-ion batteries, hard carbons still face problems, such as poor cycling performance and low initial Coulombic efficiency. Owning to the low synthesis cost and the natural presence of heteroatoms of biomasses, biomasses have positive implications for synthesizing the hard carbons for sodium-ion batteries. This minireview mainly explains the research progress of biomasses used as the precursors to prepare the hard-carbon materials. The storage mechanism of hard carbons, comparisons of the structural properties of hard carbons prepared from different biomasses, and the influence of the preparation conditions on the electrochemical properties of hard carbons are introduced. In addition, the effect of doping atoms is also summarized to provide an in-depth understanding and guidance for the design of high-performance hard carbons for sodium-ion batteries.

## 1. Introduction

At present, the depletion of natural resources, the rise of oil prices, and the various toxic gases produced from the burning of fossil fuels seriously impact the living conditions of human beings; therefore, the search for alternative green energy sources and environmental protection are becoming most urgent issues. For sustainable development, we have been focusing on the applications of clean energies such as solar energy, wind energy, tidal energy, wave energy, etc., which are not currently stable due to the climatological and environmental changes; therefore, energy storage devices are vitally important accompanying the development of green energy sources. Owing to their high theoretical capacity and good cycling performance, lithium-ion batteries (LIBs) are the most investigated and now account for 63% of the world energy storage market [1]. However, the currently detected amount of lithium (Li) metal in the Earth is only 0.0065%, which results in the high price of Li and consequently inhibits the wide application of LIBs. Apart from LIBs, sodium (Na)-ion batteries also show a relatively high capacity with a lower cost and could be regarded as the alternative to LIBs. The amount of Na reserves is 2.09%, which is about 320 times greater than that of lithium. Moreover, aluminum metal can be used as the anode current collector in Na-ion batteries, which further reduces the cost of the energy storage devices [2]. However, the Na ion radius (0.102 nm) is 0.026 nm larger than that of the Li ion (0.076 nm), so there is a gap between the required negative electrode materials for Na-ion and Li-ion batteries [3]. Currently, the anode materials of Na-ion batteries are mainly divided into metal oxides [4,5,6], metal alloys [7,8], and carbons [9]. Although the rate performance of the metal oxides is relatively good, and the theoretical capacity meets the requirements, their shortcomings are also obvious. The initial Coulombic efficiency is very low, which cannot be ignored in the practical applications. The advantage of alloys for Na-ion batteries is that the theoretical capacity is relatively high, but the volume expansion is quite serious during the reactions, resulting in poor cycling performance owing to the separation of the active materials from the electrical contact [10]. Metal sulfides can also be used in the anodes of Na-ion batteries due to their high capacity, high rate capability, and stable performance [11,12]. When considering the price, the most common negative electrodes used in batteries are carbons because they are relatively easy to obtain and many of them have porous structures, making them more suitable for the insertion and extraction of Na^+^ ions. At present, carbon-based materials applied in Na-ion batteries are mainly divided into soft and hard carbons [13]. The materials used to prepare hard carbons can be easily found, and most hard carbons have a rich microcrystalline structure, which can effectively make pathways for Na^+^ ions to shuttle as well as achieve free insertion and extraction during the charging/discharging process [14]. The so-called hard carbon cannot be graphitized even at a high temperature (1600 °C) of calcination, and the interlayer spacing is hard to change, remaining at 0.41 nm [15]. Although hard carbons are suitable for storing Na^+^ ions, there are still many problems. The biggest one is that the initial capacity is relatively low and cannot meet the commercial applications. Improving the energy density of the batteries is the priority in designing electrode materials. For example, the commonly used method is the use of MgO as a template to synthesize a hard carbon with a high capacity [16]. The most commonly researched subjects at the moment are heteroatom doping, such as with N and P atoms [17,18] and metal atoms [18,19]. Using this method, not only can the interlayer spacing be enlarged, but the initial Coulombic efficiency can be effectively improved, and consequently, the cycle stability performance of the battery can be significantly enhanced as well.

Hard-carbon materials are commonly made from phenolic resin [20]. In order to save costs, many researchers use biomasses as hard-carbon precursors [21], which are mainly derived from plant and seafood wastes [21,22] such as old loofah [23] and orange peel [24]. Plant biomasses contain a lot of lignin and cellulose; in addition to carbon, they also contain some other elements, such as N and P, which can improve the layer spacing and thus the electrochemical performance of batteries. When choosing biomasses to produce the hard carbons, we should not only consider the carbon content in the biomass, but also pay attention to the precursor structures, because the precursor is one of the main factors that directly affect the microstructures of hard carbons, which can generally be divided into linear, spherical, and porous structures. In order to meet the demands for the negative electrodes of Na-ion batteries, a porous structure is usually chosen, which is more conductive for Na^+^ ions to embed and de-embed. Most of the carbon atoms are in the form of six-membered carbon rings, but there are also pentagonal or heptagonal defect sites present. Except for graphite, the hard carbons have relatively more vacancies, edges, and defect sites, which are more suitable for elemental doping. Previous reviews have summarized usages of hard carbon [25]; however, relatively few reviews of hard carbons from biomass have been reported. The current article reviews the Na^+^ ion storage mechanism of hard carbons, summarizes the production of hard carbons using low-cost and environmentally friendly biomasses, and compares the capacity and performance of hard carbons prepared from different biomasses for Na-ion batteries.

## 2. The Mechanism of Sodium Storage in Hard Carbons

**Figure 1:**
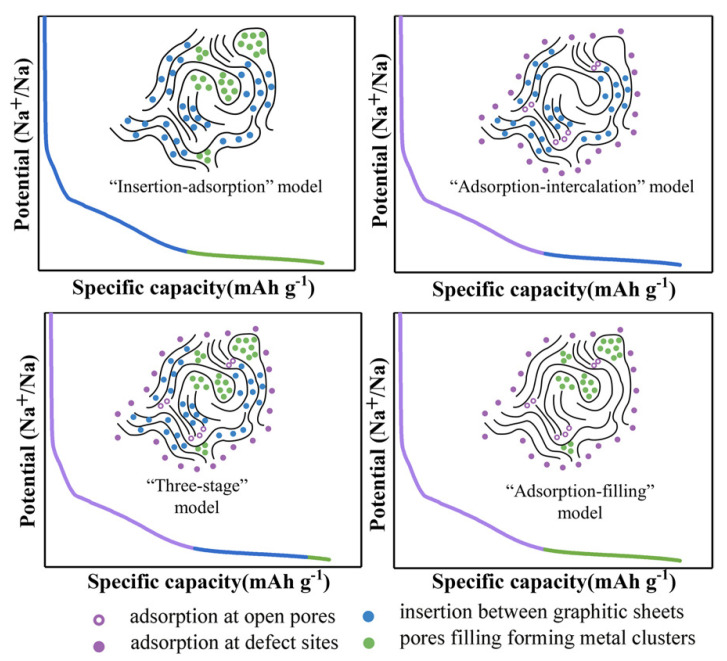
The mechanism of sodium storage in hard carbons [31], open access with copyright permission from John Wiley & Sons.

The main working principle of a Na-ion battery is based on the embedding and detachment of Na^+^ ions into and from the electrodes. Because the storage of Na^+^ ions mainly depends on the microstructure of the hard carbons, the storage mechanisms of different carbon materials are thus also expected to be different [25,26] and are divided into the following categories as shown in Figure 1. As first proposed by Stevens and Dahn in 2000, who discovered the “insertion–adsorption” mechanism using glucose as the hard carbon, its structure is similar to that of playing cards stacking, but the stacking form of carbon layers is not very orderly. A few carbon layers are parallel to each other, while most carbon layers are disorderly distributed, forming micropores between each carbon layer for the Na^+^ ions shuttling. Among them, Na^+^ ions are mainly adsorbed at the defect points and the edges of the layers at low potentials, and the ion insertion is carried out at high potentials [27]. However, some experimental results were not in line with the “insertion–adsorption” mechanism because it can disappear in the sloping area of low potential, and Na^+^ ions cannot be inserted and de-inserted, but adsorb on the surface of the carbon. Subsequently, in 2012 Cao et. al. verified the “adsorption–intercalation” mechanism of Na^+^ ions based on the data derived from x-ray diffraction (XRD); that is, in the sloping region, Na^+^ ions are adsorbed at the defective positions, while the inserted nanopores exist in the platform area. It was also confirmed that Na^+^ ion insertion into d_002_ was 0.37 nm [28]. In addition to the above two mechanisms, Bommier et al. proposed the “three-stage” (adsorption-insertion-filling) model, where Na ions are defectively adsorbed in the slope region, but in the plateau region, the Na ions are first inserted into the graphite layer and finally filled into the nanopores [29]. Then, a new mechanism was suggested via the “adsorption–insertion” model proposed by Zhang’s groups [30]. The Na ions are embedded at low potentials and depleted and adsorbed by defective sites in the high-potential regions. In addition to the above storage mechanisms, there are also some other explanations [31]. Currently, most of the experimental data now concentrate on the “adsorption–insertion” model [32].

## 3. Hard Carbons Prepared from Biomass

**Figure 2:**
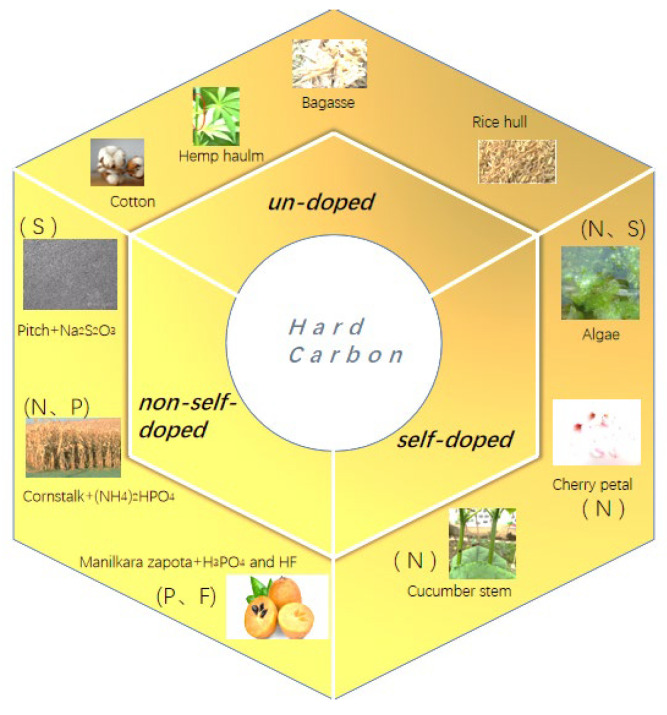
The strategy of hard carbon prepared from different biomasses.

The previous studies indicated that in addition to the low cost of biomasses, the hard-carbon materials can also be produced from the plant and seafood wastes, as the plant and seafood wastes not only occupy public space but also cause environmental pollution, this method can also achieve resource conservation, environmental protection, and sustainable development. There are many scientists and engineers who have used low-cost biomasses to produce hard carbons [33]; however, when selecting biomasses for the preparation of negative electrode materials, in addition to the high C content of the material itself, we should also pay attention to its microstructure. It is necessary to screen the microstructure of the precursor, as the different biomasses generally have different microarchitectures [34]. Figure 2 shows the preparation strategies (un-doped, non-self-doped, and self-doped) of hard carbons from different biomasses. As an example, the microstructure of the most common cotton is a linear hollow fiber structure, and the radius is about 5–10 µm.

### 3.1. Undoped Hard Carbons

In 2016, Li et al. studied the temperature impact on the hard-carbon microtubes with different pore sizes using direct pyrolysis, thereby produced a high diffusion coefficient, resulting in a good rate of performance and cycle stability. When the temperature was increased to 1300 °C, the specific surface area decreased rapidly to only 14 m^2^g^−1^; however, the reversible capacity increased significantly by more than 200 mAh g^−1^ and reached 315 mAh g^−1^ at 30 mA g^−1^, 275 mAh g^−1^ even at the current rate of 150 mA g^−1^ [35]. As the temperature increased, the number of defect sites in carbon decreased, and the quantity of oxygen-containing groups also decreased; therefore, the number of reversible Na^+^ ions increased, obtaining a higher initial Coulombic efficiency. During high-temperature pyrolysis, the structure of the hard carbon may change from disorderly to orderly and produce more graphitic carbon; it was verified by XRD that when the temperature rises from 1000 °C to 1600 °C, the diffraction peak shifts to a higher angle, indicating that the layer spacing is expanding. The number of oxygen-containing groups was reduced at a high temperature; in this case, a small specific surface area can effectively limit the formation of SEI layers and thus improve the initial Coulombic efficiency.

**Figure 3:**
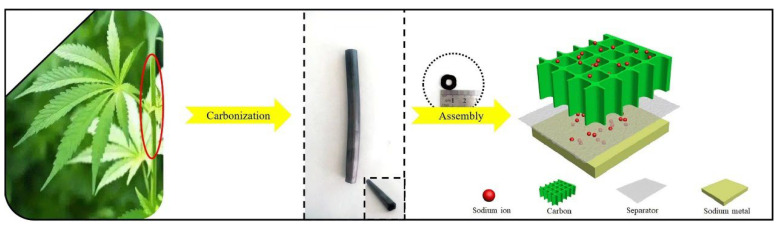
Direct pyrolysis of natural hemp haulm and assembly as a Na–ion battery [37], with copyright permission from Elsevier B. V.

The structures of the hard carbons can greatly affect the performance of the batteries. Chen et al. [36] adopted a hydrothermal method to prepare hard-carbon nanospheres from Camellia shells. After washing with strong oxidizing agents and acids, many micro-pores were created, which shortened the distance for Na^+^-ion transport. The capacity of 215 mAh g^−1^ was maintained after 100 cycles at 0.1 A/g. The three-dimensional carbon nanotube structure can make the Na^+^ ions smoothly embed and detach. As shown in Figure 3, Wang et al. adopted the microstructure of natural hemp haulm to prepare a three-dimensional hollow, straight, tubular structure [37]. After multiple washings, it was then synthesized under Ar_2_ atmosphere at 600 °C to obtain hard carbon with a pore diameter of about 10 µm. The number of carbon layers after pyrolysis was about five, and the 002 XRD peak was wider, indicating that the layer spacing (~3.97 Å) was larger than the graphite, which is more suitable for Na^+^ ion storage. The produced hard carbon showed a higher capacity in both the slope area and plateau region. The initial capacity was 256 mAh/g at a low current at 37.4 mA g^−1^, and the capacity lost only 3 mAh/g after 100 cycles, indicating excellent cycling performance. The Coulombic efficiency was close to 100% after 2000 cycles at 1870 mA g^−1^.

Sugarcane is the main raw material in sugar production. It naturally contains cellulose and lignin, but the waste generated after the sugar production is directly burned, which pollutes the environment and yields greenhouse gases [38]. When the sugarcane wastes are subjected to the pyrolysis process at 750 °C, the intensity ratio between the Raman peaks of D and G (I_D_/I_G_) is 1.33, but at 1050 °C it is 0.75, which means a 12% increase in the cycle efficiency when the temperature is increased from 750 °C to 1050 °C. After acidification, the hard-carbon surface becomes significantly rougher, and more active sites exist. For example, Hong’s group obtained many nanopores after activating pomelo peel with H_3_PO_4_, shortening the diffusion distance of the ions [39].

Pinecone is a common plant waste. It is first crushed by a kibbler, then directly carbonized at a high temperature, and then subjected to acidification and cleaned with deionized water repeatedly to obtain highly disordered hard-carbon materials. Zhang et al. found that the capacity was about 30 mAh g^−1^ higher than without washing with acid. The XRD data showed that with the increase in the carbonization temperature, the layer spacing decreased, but it still allowed ions to pass smoothly, as proved with the electrochemical impedance spectrum (EIS) results [40]. The initial efficiency reached a vertex when the pyrolysis temperature is 1400 °C.

Palm fruit is edible and can be grown in many places. The calyx of palm trees contains a high cellulose content; more important, the palm fruit possesses a hierarchical porous microstructure similar to honeycomb, which helps the infiltration of electrolytes and the removal of Na^+^ ions. With the increase in temperature, the pore size of the hard carbon produced from the palm calyx maintains a honeycomb structure of 2–10 µm, which can alleviate the problem of volume expansion during the reactions. When the temperature rises from 500 to 900 °C, the specific surface area increases by more than 500 m^2^g^−1^, which indicates that the degree of graphitization is increasing. According to the XRD data, it can be seen that with the increase in temperature, the defects in the lattice decrease. The hard carbon produced at 700 °C reaches an optimal capacity of 245 mAh g^−1^ at 50 mA g^−1^ [41]. In addition to the calyx of palm trees, Borassus flabellifer male inflorescences contain many aromatic compounds, which can also be made of low-cost hard carbons. According to the XRD results, the value of the 002 peak also increases with the increase in N_2_ and CO_2_ exhaust. When the N_2_–CO_2_ mixture ratio is 100:5, the largest porosity, suitable for ion shuttle layer spacing (0.360 nm), is obtained, which effectively increases the transport capacity of Na^+^ ions in the electrolyte and gains the highest discharge capacity of 413 mAh g^−1^, in which the charge-discharge capacity in the low potential is close, and the Coulombic efficiency in the previous cycles is close to 100% [42].

Rice is the staple food of most people. The annual rice yield is very high, but the rice husk, which is composed of lignin and cellulose, is generally used for direct combustion [43]. Hard carbon obtained through direct pyrolysis of the rice husk shows a large number of mesoporous structures because the rice husk exists in nature as a hard template of SiO_2_ [44], which improves the reversible ability of the electrode. Moreover, due to the large surface area and high porosity, the cycle stability of the obtained hard-carbon electrode is excellent: after 100 cycles at 30 mA g^−1^, the capacity loss rate is about 7%, reaching 258 mAh g^−1^ [45].

Leaves are the most abundant plant nutrition organ in the world; most of them have an anisotropic surface. The pores of leaves can provide channels for the Na^+^ ions; at the same time, the porous structure nanosheets inside the leaves overlap similarly to graphene with a low surface area, which can also improve the initial Coulombic efficiency. The specific surface area of the hard carbon produced from maple leaves is 161 m^2^/g; the large pores and low specific surface area can effectively slow down the formation of SEI and improve the initial Coulombic efficiency to 74.8% and a stable capacity of 325.1 mAh/g [46]. Lakienko et al. used summer hogweed to prepare hard carbon with an initial Coulombic efficiency of 87% and a capacity of 262 mAh g^−1^ at a current density of 25 mA g^−1^ [47].

**Figure 4:**
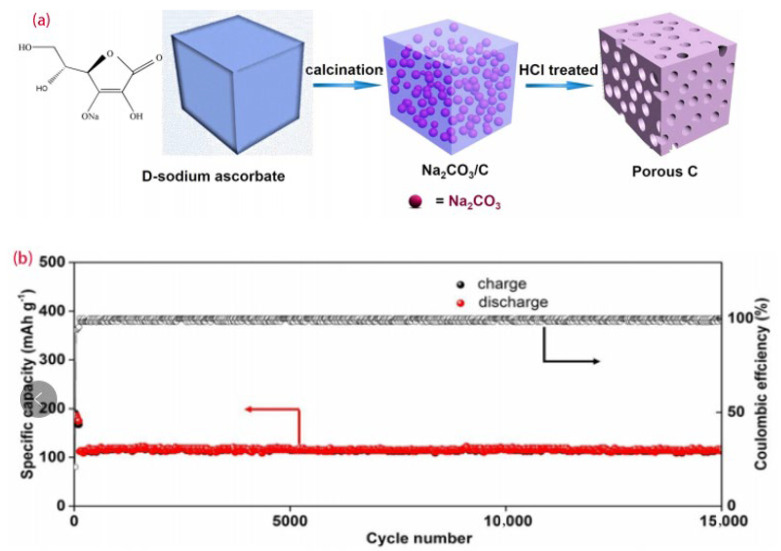
(**a**) Schematic illustration of the formation process of porous hard carbons, (**b**) long–term cycling performance of Na–ion batteries at 10 A g^−1^ [48], with copyright permission from ACS Publications.

Because the three-dimensional pores not only provide access to the electrolyte but also slow down the volume expansion during the reactions, Wang et al. adopted the non-toxic D-sodium ascorbate as the carbon source to synthesize hard carbon with a three-dimensional layered porous structure. The synthesis process is shown in Figure 4a. The battery performance was optimal with a discharge capacity of 370 mAh/g at a low current of 0.2 A g^−1^, an initial Coulombic efficiency of up to 65%, and a capacity of about 110 mAh g^−1^ at 10 A g^−1^ after 15,000 cycles, as presented in Figure 4b [48].

### 3.2. Heteroatomic Doped Hard Carbons

Heteroatom doping refers to replacing some carbon atoms or inserting other foreign atoms into the voids in the uniform carbon structure. This process provides more defects and extends the layer spacing, which significantly changes the electronic conductivity, interlamellar spacing, surface structure, and surface charge of the materials. The heteroatoms are doped into hard carbons, such as boron, nitrogen [49,50], sulfur [51], and phosphorus [52], may cause different degrees of ion and electron offset due to the different electronegativity of each element. Because their ability to adsorb to the Na^+^ ions is different from each other and from C, it tunes the charge distribution when applied in Na-ion batteries [53]. Heteroatom doping can be prepared in situ during the preparation of nanoporous carbon materials or through the post-treatment of prefabricated carbon nanomaterials with heteroatom-containing precursors [54]. The post-processing changes only the carbon surface functionalization without changing its bulk properties, whereas in situ doping can help to uniformly incorporate heteroatoms throughout the carbon matrix. When selecting biomass, in addition to paying attention to the microstructure of the precursor, it is also important whether it contains other atoms, because the doping of these atoms can effectively influence the performance of the negative electrodes. In addition, self-doping biomass can reduce a lot of experimental steps. For example, Li et al. [55] used the most common cucumber stem with a porous structure to prepare three-dimensional hard carbons with a layered mesoporous network structure via a direct pyrolysis process. The doped N and O atoms formed C-O and C-N polar bonds, through which the various pore sizes were connected to each other, effectively shortening the diffusion distance and accelerating the movement of Na^+^ ions. However, direct pyrolysis has disadvantages: there is SEI generation in the charge, reducing the initial Coulombic efficiency of un-activated hard carbons, which is 64.9% at 50 mA g^−1^. However, after KOH activation, the degree of graphitization becomes smaller, and there are more active sites to provide for the Na^+^ ions’ adsorption and desorption at the edge position, so the current density capacity increases from 337.9 to 458.6 mAh g^−1^. Moreover, the authors found that the annealing process strongly affected the properties of the hard carbons. Both the G-band and D-band of the Raman spectra were shifted to the right, and the I_D_/I_G_ values decreased with increasing temperature, indicating that the hard carbons became more disordered.

**Figure 5:**
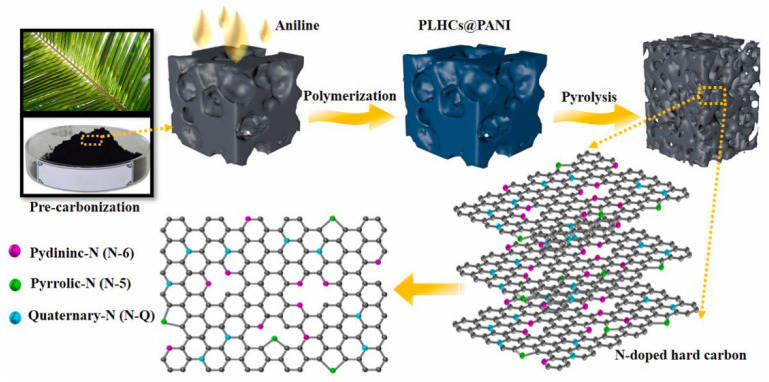
Preparation and bonding configuration of nitrogen functionalities in the N–doping palm-leaf-based hard carbon [58], with copyright permission from Elsevier B. V.

Cherry petals also naturally contain N and O atoms. Zhu et al. [56] made sheets that contained hard carbons with a large number of nanostructures via high-temperature pyrolysis and hydrochloric acid treatment to remove the impurities. The specific surface area of the hard carbons was relatively small, which not only can effectively prevent the formation of the SEI membrane, but also improve the storage capacity of Na^+^ ions. The N and O atoms were doped to expand the layer spacing of the hard carbon to 0.44 nm, which improved the specific capacity of the battery to 461.1 mAh g^−1^. Pore size analysis showed that most of the pores obtained after doping were mesoporous and macroporous, which could slow down the volume expansion in addition to increasing the number of pathways for Na^+^ ions [57]. Nie’s groups [58] used externally doped N atoms to increase the layer spacing and decrease the specific surface area. The synthesis diagram is shown in Figure 5. More important, the electrochemical performance was excellent after assembling the full cell, which delivered a discharge capacity of 78 mAh g^−1^ at a current density of 640 mA g^−1^, more than 90% of the initial capacity at a low current density at 25 mA g^−1^ after 100 cycles, and reached 373 mAh g^−1^, which is much higher than that of undoped materials.

Corn is the main food product all over the word, but corn stalks are generally directly burned, which not only wastes natural resources but also causes environmental pollution. Therefore, corn stover is very suitable as a carbon source for the synthesis of hard carbons. Qin’s group directly carbonized waste corn stover with (NH_4_)_2_HPO_4_ to produce diatom-doped hard carbon with the hydrothermal method, forming porous N, P co-doped hard carbons, which could improve the lubricity of the surface and enhance the conductivity and rate performance. XRD data showed that the spacing between the hard carbon layers after diatomic doping had expanded significantly, 0.007 nm larger than the undoped one, because of the formation of C-P, N-P bonds; pyridinic-N; and pyrrolic-N. The capacity after 200 cycles at a current density of 0.05 A g^−1^ was close to 300 mAh g^−1^. More importantly, the Coulombic efficiency was close to 100% at 0.2 A g^−1^, and the discharge capacity could remain at 122 mAh g^−1^ even at a high density of 2 A g^−1^ [59].

**Figure 6:**
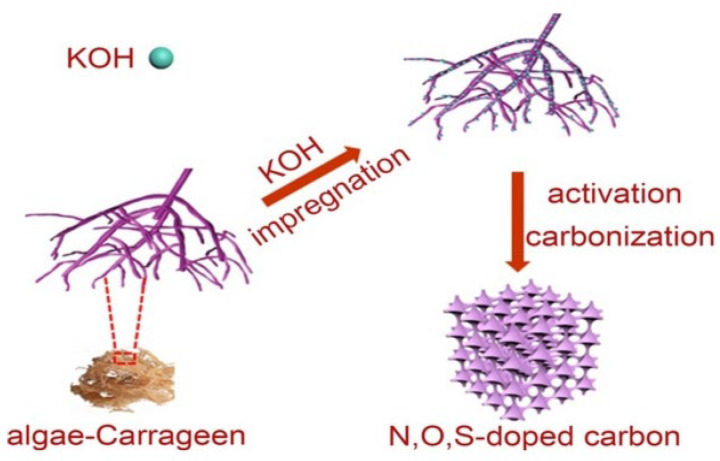
The synthesis process for the algae carrageen as hard carbon for sodium–ion batteries [51], with copyright permission from Elsevier B. V.

Carrageen contains sulfate groups, which can be directly self-doped with elements such as O, N, and S [60]. The relatively low interconnected carbon wall constructs the hard carbon with a spongy structure, and the layered porosity can be suitable for the rapid transport of Na^+^ ions. Doping of heteroatoms can inhibit the decomposition of electrolytes, reduce the loss of capacity, improve the capacity and conductivity of the hard carbons, and also accelerate the transport of Na^+^ ions. Figure 6 shows the process of synthesizing hard carbons from the algae carrageen. After activation with a KOH solution, the layer spacing increased by 0.011 nm. The capacity of the hard carbons produced from carrageen without treatment was only 104 mAh g^−1^ at 0.2 A g^−1^; the capacity increased to 248 mAh g^−1^ after the KOH solution treatment with a mass ratio to carrageen of 3:10. The capacity after doping S was higher than the doped N atoms. It was especially obvious under high current density and resulted in enhancement of the initial Coulombic efficiency to 32.7% [51]. This is because the oxidation conditions of the S element are relatively low, the binding ability of S atoms to Na atoms is strong, and the C-S-C bond is relatively stable after S doping. Because the F can solve the charge polarization to form stable C-F bonds, Wang’s group [61] calcinated the lotus petiole in Ar_2_ to produce hard carbons with a particle size below 10 µm and layer spacing of 0.4 nm. Because of the strong electronegativity of F, the repulsion between the carbon layers was increased after doping, which improved the layer spacing. The cycling performance was also significantly enhanced, and after 80 cycles, the capacity retention rate was 93% at a current density of 0.05 A g^−1^, which is much higher than those of the carbons produced from other biomasses. When the voltage was 0.001 V–2.8 V, the initial charge capacity was 230 mAh g^−1^, the initial Coulombic efficiency was 52.3% at a low rate of 200 mA g^−1^, and the charge-discharge capacity was 99.4% after 200 cycles. At a rate of 500 mA g^−1^ after 300 cycles, the capacity was 126 mA h g^−1^.

**Figure 7:**
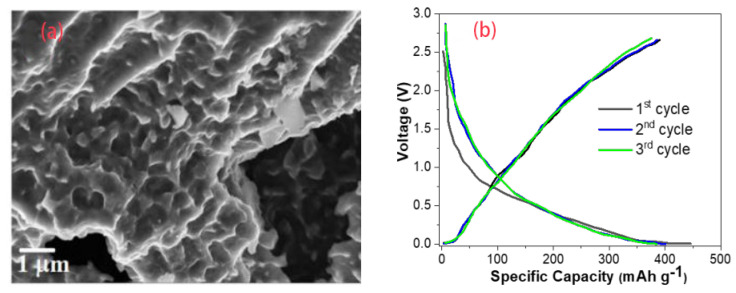
(**a**) SEM of F– and P–doping hard carbons, (**b**) the first three cycles of a Na–ion battery at 0.1 A g^−1^ current rate [64], with copyright permission from John Wiley & Sons.

Acidified biomass can provide more active sites and improve the recycling performance. Acidified hard-carbon powders from hazelnut shells not only increased the initial Coulombic efficiency to 91%, but also resulted in very little capacity [62]. Double-atom doping not only enlarged the layer spacing and increased the active sites, but also had a good synergistic effect on the storage of Na ions [63]. Dutta used the natural biomass Manilkara zapota as a precursor to produce hard carbons. The SEM image and the first three cycles are shown in Figure 7. The doping of P and F elements resulted in a significant increase in the proportion of mesopores and micropores. At a current density of 0.1 A g^−1^, the cell was able to reach a capacity of 409 mAh g^−1^, and only 6 mAh g^−1^ was lost in the first cycle [64].

Coulombic efficiency and cycling stability are important benchmarks for evaluating batteries. Table 1 is a summary of the performances of the hard carbons synthesized from various biomasses applied in Na-ion batteries. Generally, hard-carbon materials with certain morphologies have a high capacity, but most have a low initial Coulombic efficiency, which may be due to irreversible reactions that occur during the initial discharge cycle, including the formation of SEI films. Because of the unique tubular structure of cotton, which accelerates ion transport, a high capacity is achieved, and the initial Coulombic efficiency at 1300 °C can exceed 80% due to the small specific surface area. Pinecones have a high initial Coulombic efficiency (ICE) also due to their relatively small specific surface area. Natural balsa has good cycling stability and high initial capacity. Most of the hard carbons are irregularly shaped, and have high initial Coulombic efficiencies compared with the tubular structures, such as maple leaf, table sugar, and pistachio shell, etc. Another rule that can be found in Table 1 is that the synthesis temperature may strongly affect the initial Coulombic efficiency; generally, the hard carbons have higher values when produced at high temperatures.

## 4. Conclusions

Hard carbons can be produced from various biomass resources, and they display unique structures and a high capacity for Na-ion batteries. However, because of the large specific surface area, the initial Coulombic efficiency of most hard-carbon anodes is no more than 80%. This work mainly describes the progress of the preparation of hard-carbon materials and the optimization strategy in improving their initial Coulombic efficiencies. In order to achieve high-performance anodes, several criteria are required in the production of hard carbons before we take actual actions, such as the layered or columnar porous structure of the biomass precursors, which will accelerate the speed of ion transport. On the other hand, atomic doping is essential for the construction of high-energy-density Na-ion batteries, mainly because it can effectively expand the layer spacing, change the order of the hard carbon, and therefore improve the electrochemical performance. For example, after doping with common atoms like N, P, and O, etc., the cycle stability, initial Coulombic efficiency, and Na^+^ ion movement rate were significantly improved, and both the platform capacity and slope capacity increased as well.

## Figures and Tables

**Table 1:** Comparing different biomass properties of electrochemical performance.

Biomass	Temperature (°C)	Structure	Capacity (mAh g^−1^)	High-Rate Capability (mAh g^−1^)	Capacity Retention (Cycles)	ICE/%	Ref
Coconut oil	/	/	499 at 200 mA g^−1^	295 at 1000 mA g^−1^	77.7% (20)	49	[10]
Camellia shells	160	sphere	562 mAh g^−1^ at 100 mA g^−1^	123 at 1000 mA g^−1^	215 (100) at100 mA g^−1^	44.1	[36]
Hemp haulms	600	3D open	256 at 37.4 mA g^−1^	98 at 748 mA g^−1^	97.3% (100) at 37.4 mA g^−1^	/	[37]
Sugarcane	950	flake-type	290 at 0.03 A g^−1^	162 at 2 A g^−1^	94% (300) 0.1 A g^−1^	70	[38]
Pomelo peels	700	3D connected porous	180 at 200 mA g^−1^	71 at 5 A g^−1^	86.4% (220) at 200 mA g^−1^	27	[39]
Pinecones	1400	/	370 at 30 mA g^- 1^	142 at 300 mA g^−1^	90.3% (120) at 30 mA g^−1^	85.4	[40]
Palmyra palm	700	hierarchical porous network	245 at 50 mA g^−1^	131 at 500 mA g^−1^	91% (50) at 30 mA g^−1^	70	[41]
Borassus flabellifer male inflorescences	1400	/	367 at 20 mA g^−1^	117 at 2 A g^−1^	86.4% (500) at 20 mA g^−1^	86.6	[42]
Rice husks	1600	porous	276 at 30 mA g^−1^	174 at 130 mA g^−1^	93% (100) at 30 mA g^−1^	74.8	[45]
Maple leaves	1000	/	358.6 at 10 mA g^−1^	270 at 40 mA g^−1^	90% (200)	74.8	[46]
Hogweed	1300	/	221 at 25 mA g^−1^	/	95% (100) at 20 mA g^−1^	87	[47]
D-sodium ascorbate	600	3D porous	370 at 0.2 A g^−1^	126 at 20 A g^−1^	88.5% (15,000) at 10 A g^−1^	77	[48]
Pitch	/	/	870 mAh g^−1^ at 0.05 A g^−1^	145 at 5 A g^−1^	94.1% (100) at0.1 A g^−1^	56	[60]
Carrageen	700	hierarchical porous	248 at 0.2 A g^−1^	109 at 10 A g^−1^	91.5% (100) at 0.1 A g^−1^	32.7	[51]
Cucumber stems	1000	tubular porous	305.5 at 0.1 A g^−1^	136.6 at 5 A g^−1^	82.1% (500) at 0.2 A g^−1^	64.9	[55]
Cherry petals	1000	open lamellar	298 at 20 mA g^−1^	146.5 at 500 mA g^−1^	99.3% (100) at 20 mA g^−1^	67.3	[56]
Palm leaves	1000	parallel columnar	373 at 25 mA g^−1^	298 at 100 mA g^−1^	95% (100) at 200 mA g^−1^	44.2	[58]
Corn stalks	1200	folded lamellar	241 at 50 mA g^−1^	103 at 1 A g^−1^	79% (70) at 1 A g^−1^	53.1	[59]
Lotus petioles	1400	/	230 at 50 mA g^−1^	158 at 1 A g^−1^	99.1% (200) at 200 mA g^−1^	52.3	[61]
Manilkara zapota	500	/	378 at 100 mA g^−1^	287 at 1.5 A g^−1^	93.1% (1000) at 0.1 A g^−1^	87.5	[64]
Banana peels	1100	heterogeneous	355 at 50 mA g^−1^	155 at 1 A g^−1^	88% (290) at 100 mA g^−1^	67.8	[65]
Table sugar	1200	/	213 at 20 mA g^- 1^	Over 100 at 0.5 A g^−1^	89% (100) at 20 mA g ^−1^	89	[66]
Pistachio shells	1000	/	225 at 10 mA g^−1^	about 100 at 200 mA g^−1^	91.5% (50) at 40 mA g^−1^	71	[67]
Lotus stems	1400	/	351 at 40 mA g^−1^	230 at 500 mA g^−1^	94.2% (450) at 100 mA g^−1^	70	[68]
Peanut shells	600	porous	325 at 100 mA g^−1^	about 150 at 1000 mA g^−1^	86% (400) at 250 mA g^−1^	/	[69]
Natural balsa	1200	3D channel pore	439 at 100 mA g−1	215 at 2 A g^−1^	93.5% (500) at 2 A g^−1^	31.9	[70]
Glucose	1100	spheres	160 at 50 mA g^−1^	/	160 mAh g^−1^ (200) at 50 mA g^−1^	/	[71]
Tea leaves	1000	spheres	375.3 at 100 mA g^−1^	142 at 1 A g^−1^	79% (200) at 1000 mA g^−1^	61.4	[72]
Sorghum stalks	1300	/	245 at 20 mA g^−1^	172 at 200 mA g ^−1^	96% (50) at 20 mA g^−1^	62.2	[73]

## Data Availability

Not applicable.

## Sample Availability

Samples of the compounds hard carbons are available from the authors.
